# Workers’ Memorial Day — April 28, 2014

**Published:** 2014-04-25

Workers’ Memorial Day, observed on April 28, 2014, recognizes workers who died or suffered from exposures to hazards at work. In 2012, a total of 4,383 U.S. workers died from work-related injuries (1). Most fatalities from work-related illness are not captured by national surveillance systems, but an estimate for 2007 was 53,445 deaths (2).

In 2012, nearly 3 million injuries to and illnesses in private industry workers and 793,000 to and in state and local government workers were reported by employers (3). In the same year, an estimated 2.8 million work-related injuries were treated in emergency departments, resulting in 140,000 hospitalizations (National Institute for Occupational Safety and Health, CDC, unpublished data, 2014). Several national surveillance systems report new cases of nonfatal work-related injuries and illnesses, although no system captures all cases. Based on methods that focus on medical costs and productivity losses, the societal cost of work-related fatalities, injuries, and illnesses was estimated at $250 billion in 2007 (2). Methods that include consideration of pain and suffering would result in a higher estimated societal cost (4). CDC is working to better describe the burden of fatalities, injuries, and illnesses suffered by workers; additional information is available at http://www.cdc.gov/niosh/programs/econ/risks.html.

## References

[1] Bureau of Labor Statistics (2013). National Census of Fatal Occupational Injuries in 2012 preliminary results: Table 2.

[2] Leigh JP (2011). Economic burden of occupational injury and illness in the United States. Millbank Q.

[3] Bureau of Labor Statistics (2013). Employer-reported workplace injuries and illnesses in 2012. Table 2.

[4] Haddix AC, Teutsch SM, Corso PS (2003). Prevention effectiveness: a guide to decision analysis and economic evaluation.

